# Daily almond consumption in cardiovascular disease prevention via LDL-C change in the U.S. population: a cost-effectiveness analysis

**DOI:** 10.1186/s12889-020-08642-4

**Published:** 2020-04-25

**Authors:** Jifan Wang, Michelle A. Lee Bravatti, Elizabeth J. Johnson, Gowri Raman

**Affiliations:** 1 Tufts University Friedman School of Nutrition and Policy, 150 Harrison Ave, Boston, MA 02111 USA; 2 Tufts Clinical Evidence Synthesis Center, Tufts Medical Center, 800 Washington Street, box 63, Boston, MA 02111 USA

**Keywords:** Almond, Cost-effectiveness analysis, Cardiovascular disease, Heart disease, Stroke, Myocardial infarction

## Abstract

**Background:**

Heart disease is the leading cause of death in the United States. The U.S. Food and Drug Administration approved the health claim that 1.5 oz (42.5 g) of nut intake may reduce the risk of cardiovascular disease. Previous studies have focused on the cost-effectiveness of other foods or dietary factors on primary cardiovascular disease prevention, yet not in almond consumption. This study aimed to examine the cost-effectiveness of almond consumption in cardiovascular disease primary prevention.

**Perspective & Setting:**

This study assessed the cost-effectiveness of consuming 42.5 g of almond from the U.S. healthcare sector perspective.

**Methods:**

A decision model was developed for 42.5 g of almond per day versus no almond consumption and cardiovascular disease in the U.S. population. Parameters in the model were derived from the literature, which included the probabilities of increasing low-density lipoprotein cholesterol, developing acute myocardial infarction and stroke, treating acute myocardial infarction, dying from the disease and surgery, as well as the costs of the disease and procedures in the U.S. population, and the quality-adjusted life years. The cost of almonds was based on the current price in the U.S. market. Sensitivity analyses were conducted for different levels of willingness-to-pay, the probabilistic sensitivity analysis, ten-year risk prevention, different costs of procedures and almond prices, and patients with or without cardiovascular disease.

**Results:**

The almond strategy had $363 lower cost and 0.02 higher quality-adjusted life years gain compared to the non-almond strategy in the base-case model. The annual net monetary benefit of almond consumption was $1421 higher per person than no almond consumption, when the willingness to pay threshold was set at $50,000 for annual health care expenditure. Almond was more cost-effective than non-almond in cardiovascular disease prevention in all the sensitivity analyses.

**Conclusion:**

Consuming 42.5 g of almonds per day is a cost-effective approach to prevent cardiovascular disease in the short term and potentially in the long term.

## Background

Almonds contain a variety of bioactive components that have been individually related to cardiovascular health [1]. Almonds, along with other tree nuts, are good sources of mono- and polyunsaturated fats that have been shown to lower blood lipid levels. Although there is no direct study investigating the effect of almond on cardiovascular disease outcomes, our recent meta-analysis found that almond consumption reduced the level of cardiovascular disease (CVD) risk factors, such as low-density lipoprotein cholesterol (LDL-C), total cholesterol, body weight, and apolipoprotein B [2]. The qualified health claim for tree nuts and heart health by the U.S. Food and Drug Administration states, “Scientific evidence suggests but does not prove that eating 1.5 ounces per day of most nuts, as part of a diet low in saturated fat and cholesterol, may reduce the risk of heart disease” [3].

Cardiovascular disease treatments are usually expensive, which include medications and invasive or non-invasive surgeries. Between 2014 and 2015, the estimated direct cost of CVD and stroke was $213.8 billion in the U.S [4]. Although some studies have been conducted to assess the cost-effectiveness of those treatments, [5] findings suggest that treatments such as statin medication are effective, but can have side-effects on health [6].

In contrast, tree nuts as part of a healthy diet, typically do not have any side effects on consumers, with the exception of tree nut allergies [7]. Given the fact that nuts, including almonds, are relatively expensive, it is not clear whether consuming almonds on a daily basis would be a cost-effective way to prevent CVD. The purpose of this research is to determine whether the consumption of almonds is an economically preferred alternative for CVD primary prevention using both short-term base case analysis and 10-year risk prevention.

## Methods

### Target population and study perspective

The target population of this study is U.S. adults with increased risk of type 2 diabetes, including overweight or obese, or normal-weight adults with a strong family history of diabetes, based on the original intervention study we used for the analysis. The mean age of participants in the original study was approximately 30 years in both almond and non-almond group with an average body mass index (BMI) of no less than 27 kg/m^2^. The randomized control trial recruited 150 participants, of which 48 men and 89 women completed all study activities. Each of the five arms in the study had similar sex ratios [8]. This current study applied the healthcare sector’s perspective to inform individual decisions on using daily almond consumption for CVD primary prevention.

### Base-case decision model

**Fig. 1:**
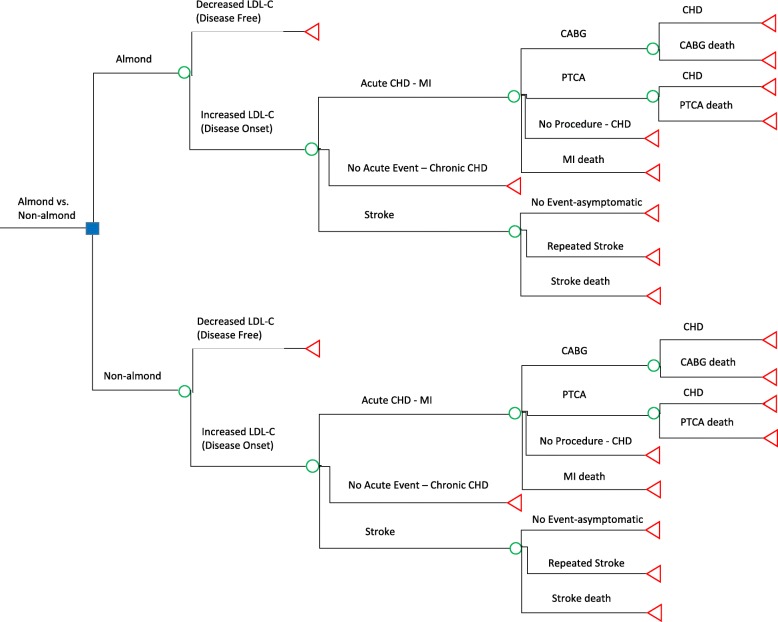
Decision-making model. The blue square is the decision node. Green circles are chance nodes. Red triangles are terminal nodes. CABG = coronary artery bypass graft; CHD = chronic heart disease; LDL-C = low-density lipoprotein cholesterol; MI = myocardial infarction; PTCA = percutaneous transluminal coronary angioplasty

We developed a decision model for CVD primary prevention among adults with 42.5 g of (1.5 oz) almond consumption per day (almond strategy), as compared with no almond consumption (non-almond strategy) to project 1-year health outcomes and CVD-related costs (Fig. 1). Previous studies on statin have shown that 1 year could be sufficient for CVD primary prevention [9]; therefore, we chose to use 1 year for our base-case analysis and to further assess the long-term effect in the sensitivity analysis. We referred to a previous paper to develop the model structure [10]. Our previous meta-analysis found a significant decrease in LDL-C among almond intervention groups, as compared with no almond controls [2]. Level of LDL-C was applied as the determinant for possible risk for future CVD events. Individuals with lower or normal levels of LDL-C, who did not have CVD, started in the “disease-free” health state, either in the almond or non-almond strategy. We assumed that all the probabilities of CVD events were the same in the almond and non-almond strategy if their LDL-C increased. The probabilities of changes in LDL-C for the almond and non-almond strategy were obtained through contact with the study authors [8]. Transitions from the “disease-onset” health state to CVD events, including acute myocardial infarction (MI), stroke, and subsequent procedures or outcomes were based on probabilities derived from targeted literature reviews (Table 1). The probabilities of developing outcomes in the one-year time frame were converted from the original data to rates and then to probabilities according to the following equations, [28] assuming that the risk was the same every year:

$$ r=\frac{-\ln \left(1-p\right)}{t}; $$

$$ Probability=1-{\mathit{\exp}}^{- rt\prime } $$

where *r* is the rate from original data; *p* is the original probability for the time frame in the literature; *t* is the original study duration; *t’* is the time frame in the analysis, equaling one in our base-case model and ten in the 10-year risk prevention model.

**Table 1:** Input Parameters in the Decision-making Model and Source

Parameter	Value	Distribution	Source
Probability			
Increase in LDL-C	29% (almond)	–	Tan et al. [8]^a^
	41% ± 12% (almond)	Beta	Tan et al.[8]^a^
	44% (non-almond)	–	Tan et al.[8]^a^
For CVD patients	25% (almond)	–	Chen et al. [11]
	35% (non-almond)	–	Chen et al. [11]
Developing MI	0.38%	–	Pikula et al. [12]
	3.75%*	–	Pikula et al. [12]
	1.04% ± 0.91%	Beta	Multiple sources [12, 13]
Death due to MI	14%	–	Benjamin et al. [14]
Taking CABG	0.11%	–	Epstein et al. [15]
Death due to CABG	1.85%	–	Eisenberg et al. [16]
Taking PTCA	0.37%	–	Epstein et al. [15]
Death due to PTCA	1.82%	–	Benjamin et al. [14]
Developing stroke	0.25%	–	Pikula et al. [12]
	2.46%*	–	Pikula et al. [12]
	0.099% ± 0.11%	Beta	Multiple sources [12, 17, 18]
Recurrent stroke	30.33%	–	Benjamin et al. [14]
Death due to stroke	21.82%	–	Benjamin et al. [14]
Cost (in 2012 USD)			
Almond	$156^b^	–	Trader Joe’s [19]
	$1369*	–	Trader Joe’s [19]
Organic almond	$470	–	US market price [20]
CABG Procedure	$37,448	–	Cohen et al. [21]
Sensitivity	$29,609	–	Caruba et al. [22]
Follow-up of CABG	$6918	–	Cohen et al. [21]
	$60,548*	–	Cohen et al. [21]
Failure to Rescue after CABG	$5733^c^	–	Cohen et al. [21]
PCI Procedure	$31,036	–	Cohen et al. [21]
Sensitivity	$13,688	–	Caruba et al. [22]
Follow-up of PCI	$9489	–	Cohen et al. [21]
	$83,050*	–	Cohen et al. [21]
Failure to Rescue after PCI	$9243^c^	–	Cohen et al. [21]
Treatment to Acute MI	$14,697	–	Cohen et al. [21]
Treatment to Chronic Heart Disease	$3365	–	Caruba et al. [22]
	$45,709*	–	Caruba et al. [22]
Recurrent Stroke	$61,988	–	Engel-Nitz et al. [23]
	$330,528*	–	Engel-Nitz et al. [23]
Death due to Stroke	$11,377	–	Russo & Andrews [24]
Utilities			
Disease free	1 QALY	–	–
	8.75 QALYs*	–	–
Successful CABG	0.82 QALY	–	Elizabeth et al. [25]
	7.14 QALYs*	–	Elizabeth et al. [25]
Successful PCI	0.85 QALY	–	Elizabeth et al. [25]
	7.44 QALYs*	–	Elizabeth et al. [25]
Chronic Heart Disease	0.86 QALY	–	Bakhai et al. [26]
	7.53 QALYs*	–	Bakhai et al. [26]
Recurrent Stroke	0.48 QALY	–	Nelson et al. [27]
	4.20 QALYs*	–	Nelson et al. [27]
Death	0 QALY	–	–

Abbreviations: CABG = coronary artery bypass graft; LDL-C = low-density lipoprotein cholesterol; MI = myocardial infarction; PCI = percutaneous coronary intervention; PTCA = percutaneous transluminal coronary angioplasty. Note that PTCA and PCI were used interchangeably in data collection. ^a^Data is obtained from the request to author. ^b^Cost of almond was calculated based on the price of $4.99/lb. and consuming 42.5 g almond every day. ^c^Failure to rescue after procedures includes the cost of re-hospitalizations, physician fees, outpatient services, and medication cost. *Highlighted data was used in the 10-year model

After an acute MI event, health states were further classified as: 1) undergoing a procedure (coronary artery bypass graft (CABG), percutaneous transluminal coronary angioplasty (PTCA)), 2) no procedure (but managed medically), 3) having a MI-related death. After an event of stroke, health states were further classified as: 1) asymptomatic stroke, 2) recurrent stroke, and 3) death from stroke. Once in a CVD disease state, individuals could not transition back to a “disease-free” state. After an acute disease state, individuals transitioned to a chronic heart disease (CHD) state.

### Cost of therapy

The cost of almonds was derived from a publicly available source as the current price of almonds in the U.S. market [19]. The annual cost was calculated based on the consumption of 42.5 g per day. The costs of CVD events and costs of treatments were derived from recent literature [21–24]. The costs of each procedure (i.e., CABG or PTCA) included procedural and physician fees as well as costs for hospital stays and ancillary services. For procedures following the CHD state, we considered costs for re-hospitalization, outpatient and rehabilitation services, medication, and physician fees [22]. The costs for medical therapy and emergency admission for MI were used for the “no procedure” outcome. For the costs for direct death due to MI, we included physician fees, hospital stay expenses and ancillary services [21]. We used the first-year follow-up costs for stroke medication and rehabilitation as the cost for recurrent stroke [23]. The five-day hospitalization cost for cerebrovascular disease was used as the cost for death from stroke since the average cost and the length of stay is similar between the two events [24]. All the costs were adjusted to 2012 U.S. dollars, the year when the almond randomized controlled trial (RCT) [8] was conducted. Medical expenditure was adjusted using the Personal Health Care Index [29] and the almond cost was adjusted using Consumer Price Index [30].

### Quality-adjusted life year

Quality-adjusted life year (QALY) for each outcome was used as the effectiveness in the model. We assumed that the QALY of the disease-free stage was equal to 1. All input parameters in the model are listed in Table 1.

### Cost per quality-adjusted life year threshold

We used multiple cost-effectiveness thresholds based on resources available for the typical U.S. decision maker [31]. The threshold of $50,000-per-QALY was used as the lower boundary, which has been the ratio established by the U.S. government in 1970s that mandates Medicare coverage for end-stage renal disease (ESRD) patients [32]. The threshold of $100,000-per-QALY was used as the willingness to pay (WTP) of twice the per capital annual income of $54,000, which has been suggested by economists and the World Health Organization (WHO) as a reasonable threshold based on empirical estimates and economic theory [32]. The highest threshold of $200,000-per-QALY was based on the increase in health spending over time and surveys asking people about their WTP in exchange of health gains [33, 34].

### Sensitivity analyses

We performed several one-way sensitivity analyses, in which the cost-effectiveness ratio was calculated by altering the following parameters identified from targeted literature reviews: 1) the probabilities of developing CVD in 10 years; 2) the costs of CABG and PTCA procedures; 3) the cost of almonds; and 4) the LDL-C response among participants with existing CVD. In the 10-year model, we applied a 3% per year discount rate to costs and effectiveness [35, 36].

We further conducted Monte Carlo probabilistic sensitivity analysis (PSA) with 10,000 simulations to address uncertainty. We extracted data from Pikula et al. and other literature [13, 17, 18] to estimate the distributions of key (parameters)

Preferred alternative was chosen based on the net monetary benefit (NMB):

$$ \hat{NMB}=\lambda \times \Delta \overline{E}-\Delta \overline{C} $$

where λ is the maximum WTP for health care, ∆$\overline{E}$is the difference in the mean effectiveness of two strategies, and ∆$\overline{C}$is the difference in the mean cost of two strategies [37]. TreeAge Pro 2018 was used to conduct the analyses.

## Results

### Base-case decision model

The base-case decision model for 1 year showed that consuming 42.5 g of almonds per day was a preferable strategy to prevent CVD outcomes such as MI, CHD, and stroke (Table 2). The results showed that 42.5 g of almond consumption every day costs an individual $1211/QALY and no almond consumption costs $1625/QALY. The annual NMB was $46,794 and $45,373 for the almond and non-almond strategy, respectively, when the WTP was $50,000 for individual health care expenditure every year. A negative incremental cost-effectiveness ratio (ICER) was obtained due to lower costs of almond consumption in relation to the higher amount of QALYs gained; therefore, the non-almond strategy was dominated. When the WTP was increased to $100,000 and $200,000, the NMB of almond strategy correspondingly increased to $94,749 and $190,658 while the NMB of non-almond increased to $92,270 and $186,064; the almond strategy always had a higher NMB than the non-almond strategy regardless of the WTP.

**Table 2:** Results of Decision Model and Sensitivity Analyses

	Cost ($)	ΔC ($)	Outcome (QALYs)	ΔE (QALYs)	C/E ($/QALY)	ICER ($/QALY)	NMB ($)
Decision model							
*WTP = $50,000*							
Non-almond	1524	Ref	0.94	Ref	1625	Dominated	45,373
Almond	1161	−363	0.96	0.02	1211		46,794
*WTP = $100,000*							
Non-almond	1524	Ref	0.94	Ref	1625	Dominated	92,270
Almond	1161	−363	0.96	0.02	1211		94,749
*WTP = $200,000*							
Non-almond	1524	Ref	0.94	Ref	1625	Dominated	186,064
Almond	1161	−363	0.96	0.02	1211		190,658
Sensitivity–Probabilistic Sensitivity Analysis							
Non-almond	1555 ± 59	Ref	0.94 ± 0.0005	Ref	1658 ± 63		45,333 ± 84
Almond	1589 ± 417	34 ± 414	0.94 ± 0.02	0.005 ± 0.02	1694 ± 474	−26,798 ± 814,514	45,542 ± 1245
Sensitivity–10-year model							
Non-almond	20,871	Ref	8.13	Ref	2566	Dominated	385,788
Almond	15,120	−5750	8.37	0.24	1806		403,377
Sensitivity–cost of procedure							
Non-almond	1524	Ref	0.94	Ref	1625	Dominated	45,373
Almond	1161	−363	0.96	0.02	1210		46,794
Sensitivity–cost of almond							
Non-almond	1524	Ref	0.94	Ref	1625	Dominated	45,373
Higher cost of almond	1474	−50	0.96	0.02	1537		46,480
Sensitivity–CVD patients							
Non-almond	1213	Ref	0.86	Ref	1411	Dominated	41,766
Almond	1022	−190	0.86	0.0001	1189		41,962

Abbreviations: *C/E* cost-effectiveness ratio, *ICER* incremental cost-effectiveness ratio, *NMB* net monetary benefit, *QALY* quality-adjusted life years, *WTP* willingness-to-pay

### Sensitivity analyses

**Fig. 2:**
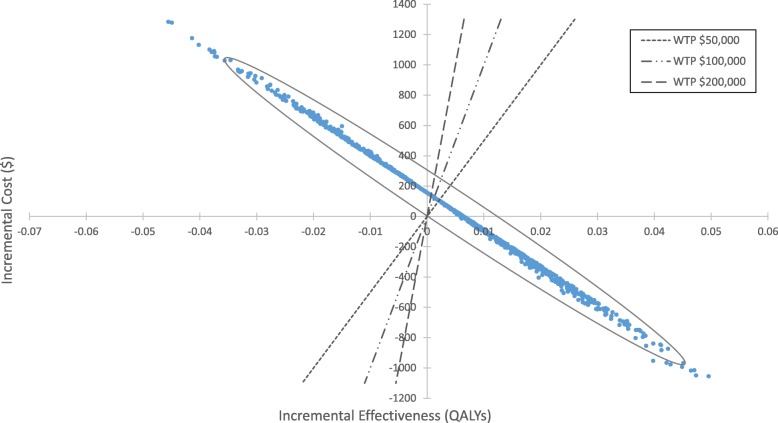
Scatter plot of estimated incremental cost ($) and incremental effectiveness (QALYs) of 1.5 oz almond versus non-almond from the probability sensitivity analysis. Dashed lines denote the willingness-to-pay (WTP) at $50,000, $100,000, and $200,000, respectively. The area to the right of the WTP indicates the almond strategy being cost-effective

**Fig. 3:**
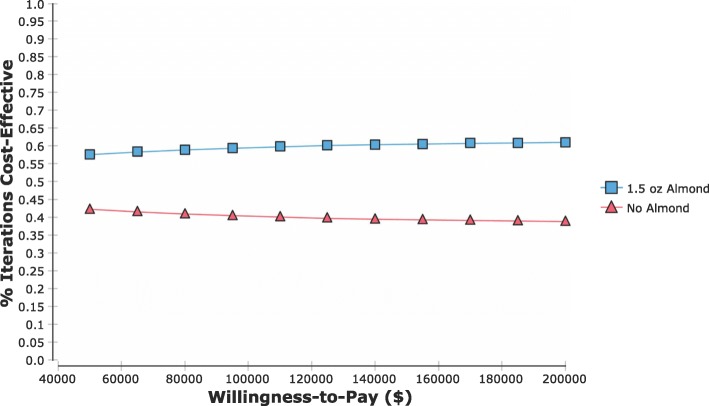
Cost-effectiveness accessibility curve (CEAC) of multiple thresholds for willingness-to-pay (WTP). Red triangles depict no almond; blue squares depict 1.5 oz almond. The WTP for health care ranged from $50,000-per-QALY to $200,000-per-QALY. The CEAC shows the probability of a strategy being the more cost-effective alternative under different thresholds of WTP

In the PSA, on average, almond strategy had $34 ± 414 increased cost and 0.005 ± 0.02 increased QALY compared to non-almond strategy. The NMB for consuming almond was $45,542 ± 1245 while the NMB for non-almond was $45,333 ± 84. The almond strategy had a 58, 60, and 61% probability of being cost-effective at the WTP of $50,000, $100,000, and $200,000, respectively (Figs. 2 and 3).

When we expanded the time horizon to 10 years, the non-almond strategy was still dominated as it had a higher cost, but a lower effectiveness compared to the almond strategy (Table 2). The almond strategy cost $5750 less, but gained 0.24 QALYs more than the non-almond strategy. The NMB for almond was $17,589 higher than the non-almond strategy.

In other sensitivity analyses (Table 2), the non-almond strategy continued to be dominated even when different costs of procedures were input. The results from different procedure costs remained the same as the results from the base-case model. As the price of almond increased, it cost more money per QALY to prevent CVD by consuming almonds; however, it was still more financially viable than not consuming almonds. With the price of organic almonds, it cost $1537/QALY for almond consumption with an NMB of $46,480.

For secondary prevention, the almond strategy cost $1189/QALY compared with $1411/QALY for the non-almond strategy, and had a higher NMB ($41,962 for almond vs. $41,766 for non-almond).

## Discussion

This study assessed the cost-effectiveness of almond consumption in the short term and up to 10 years for CVD prevention. We found that it costs an individual $1211/QALY to prevent CVD in 1 year by consuming almonds everyday versus $1625/QALY for no almond, indicating that consuming almonds may be cost-effective to prevent CVD in the short term. It cost $1806/QALY for almond versus $2566/QALY for no almond in 10-year CVD primary prevention; therefore, consuming almonds may be potentially cost-effective in the long term. In the sensitivity analyses, consuming almonds was also a financially viable way to prevent CVD. The non-almond strategy was dominated in almost all sensitivity analysis except in the PSA.

Heart disease is the leading cause of death in the United States, with over 630,000 deaths in 2015 and over 140,000 stroke-related deaths in the same year [38]. The disease also lays a huge economic burden in the United States. Between 2014 and 2015, the estimated annual cost of CVD in the United States was $351.2 billion. The projected total costs of CVD until 2035 will continue to increase for people in all age groups [4]. Under such disease and economic burden, cost-effective primary prevention strategies for CVD are imperative for the population.

Almonds have been studied continuously due to its cardiovascular benefits. Our recent meta-analysis showed a reduction in CVD risk factors, such as LDL-C, total cholesterol, body weight, and apolipoprotein B with almond consumption, with no difference on triglycerides, blood pressure, apolipoprotein A1, high-sensitivity C-reactive protein, and lipoprotein (a) [2]. Almonds contain phytochemicals such as proanthocyanidins, hydrolysable tannins, fat-soluble bioactives including vitamin E and phytosterols, and antioxidants that are cardio-beneficial. Other macro- and micro-nutrient components in almonds, including omega-3 fatty acids, selenium, magnesium, copper, potassium, and β-sitosterol, are also potentially cardio-protective [1].

Previous studies have focused on the cost-effectiveness of other foods or dietary factors on primary CVD prevention, [39–42] but little is known about the cost-effectiveness of almonds or other tree nuts. A recent study assessed the effect of healthy food financial incentives from both societal and healthcare perspectives, showing that 30% subsidy on healthy food, including nuts, is a cost-effective way to prevent CVD and diabetes [43]. To our best knowledge, our study is the first cost-effectiveness research on CVD primary prevention using an almond strategy at the individual level. In this study, we conducted a base-case model and several sensitivity analyses to assess the cost-effectiveness in the short term and the long term. The results of this study may provide some insights on individual level healthy dietary behaviors as well as population level benefits of consuming almonds.

Our study is mainly constrained by lack of available data. We derived the probabilities of developing MI and stroke from Pikula et al. in which participants had older age, higher total cholesterol levels, and 9% diabetes, but similar sex ratio and high-density lipoprotein levels compared to our target population. Although the populations were not completely matched for all CVD risk factors, Pikula et al. was the most appropriate study that provided the probabilities for two of the key parameters for the base-case model [12]. We used LDL-C response as our mediator of CVD risk, even though the ratio of total cholesterol/high-density lipoprotein may have been a better indicator as it reflects both benefits and side effects of almonds; however, we were only able to obtain the data of LDL-C response from study authors. Due to the wide variety of health insurance options in the United States, we were not able to summarize the average premiums, deductibles, and out-of-pocket expenses for CVD treatments. Instead, we used the average healthcare cost for each treatment; consequently, our results and conclusion may only apply to the uninsured. Regarding the parameters in the PSA, we were only able to find limited data that had a different center of distribution to estimate the uncertainty of the results.

Our models had a few other limitations. For example, the focus of this study was on the U.S. population with an increased risk of type 2 diabetes using the costs of medical treatments and the probabilities of developing diseases from studies conducted in the United States. Therefore, the results may not be generalizable to populations in other countries. Furthermore, unlike medical or surgical therapies, there are no serious side effects for consuming almonds, except for tree nut allergies. Thus, our models do not take into consideration any serious side effects, which could be related to the preference of the almond strategy.

The interpretation of our results requires more caution. First, our study was based on inputs from published literature instead of primary data from an intervention cohort. Thus, the inputs were constrained by the study design of the literature, especially the probabilities. As a result, we made three assumptions in the models: 1) changes in LDL-C can lead to a difference in CVD risk in one year in the base-case model; 2) changes in LDL-C caused by almonds remained consistent in the ten-year sensitivity analysis; and 3) costs of almonds and procedures over time remained consistent in the ten-year sensitivity analysis. More data may be needed to estimate the costs of almonds and procedures over time.

## Conclusion

Consuming almonds 42.5 g per day is a cost-effective approach to prevent CVD in the short term and potentially up to 10 years. Given the fact that the American population consumed an average of 2.93 g of almonds daily in the 2017–2018 crop year, [44] the potential benefits of increasing the almond consumption to the recommended level could be significant.

## Data Availability

The data were publicly available in published articles or were obtained through request from Dr. Tan [8] and Dr. Chen [11]. The data used and analyzed during the current study is available from the corresponding author on reasonable request.

## Abbreviations

CABG: coronary artery bypass graft
CHD: chronic heart disease
CVD: cardiovascular disease
ICER: incremental cost-effectiveness ratio
LDL-C: low-density lipoprotein cholesterol
MI: myocardial infarction
NMB: net monetary benefit
PSA: probabilistic sensitivity analysis
PTCA: percutaneous transluminal coronary angioplasty
QALY: quality-adjusted life years
